# Albumin Restores Endothelial Cell Mitochondrial Morphology in Patients With Decompensated Cirrhosis

**DOI:** 10.1111/liv.70653

**Published:** 2026-04-20

**Authors:** Susan E. Fischer, Rudmer J. Postma, Roel Bijkerk, Annarein J. C. Kerbert, Anton Jan van Zonneveld, Minneke J. Coenraad

**Affiliations:** ^1^ Department of Gastroenterology and Hepatology Leiden University Medical Centre Leiden the Netherlands; ^2^ Department of Internal Medicine (Nephrology) and the Einthoven Laboratory for Vascular and Regenerative Medicine Leiden University Medical Centre Leiden the Netherlands

**Keywords:** decompensated cirrhosis, endothelial cell activation, hypoalbuminemia, mitochondrial function, mitochondrial morphology

## Abstract

**Background & Aims:**

Endothelial cell (EC) activation is a critical driver of disease progression in cirrhosis, contributing to acute decompensation (AD) and acute‐on‐chronic liver failure (ACLF). Although human albumin administration improves EC function in cirrhotic patients with hypoalbuminemia, its direct effects on ECs remain unclear. This ex vivo study investigates the impact of albumin on EC morphology and mitochondrial function upon exposure to plasma from patients with decompensated cirrhosis (DC).

**Methods:**

Human umbilical vein ECs were exposed to plasma from patients with DC and hypoalbuminemia (albumin < 30 g/L, *n* = 20), compensated cirrhosis (CC, > 30 g/L, *n* = 20), or healthy controls (HC, *n* = 20). Albumin was added to DC and HC plasma to reach physiological (~40 g/L) or supraphysiological levels. Mitochondrial function was assessed by measuring oxygen consumption rate (OCR) and reactive oxygen species (ROS) production. The effects of albumin on EC activation were tested using circulating factors elevated in DC (lipopolysaccharide (LPS), tumour necrosis factor‐α (TNFα), bilirubin).

**Results:**

Mitochondrial morphology distinguished ECs exposed to DC plasma from those exposed to CC or HC plasma. Albumin supplementation shifted EC morphology towards a healthier phenotype. ECs exposed to DC plasma showed increased mitochondrial respiration without a concomitant increase in ROS production, which was normalised by albumin. Albumin had no significant effects on EC activation induced by circulating factors.

**Conclusions:**

Plasma from patients with DC and hypoalbuminemia induces EC morphological changes, particularly in mitochondria. Albumin mitigates these effects, suggesting a direct modulatory role on mitochondrial function and supporting its therapeutic potential in vascular dysfunction in cirrhosis.

## Key Points

- Plasma from patients with decompensated cirrhosis and hypoalbuminemia induces changes in endothelial cell morphology.
- Albumin administration restores EC morphological changes, specifically mitochondrial morphology and function.
- Targeting endothelial dysfunction may help mitigate complications in advanced liver disease.

**Abbreviations**
ACLF: Acute‐on‐Chronic Liver Failure
AD: Acute Decompensation
ATP: Adenosine Triphosphate
BSA: Bovine Serum Albumin
CaCl₂: Calcium Dichloride
CC: Compensated Cirrhosis
CLIF‐AD: Chronic Liver Failure Consortium Acute Decompensation
DAMPs: Damage‐Associated Molecular Patterns
DC: Decompensated Cirrhosis
EBM: Endothelial Cell Basal Medium
EC: Endothelial Cell
ECAR: Extracellular Acidification Rate
EDTA: Ethylenediaminetetraacetic Acid
EF‐CLIF: European Foundation for the Study of Chronic Liver Failure
EGM: Endothelial Cell Growth Medium
FCCP: Carbonyl cyanide‐4‐(trifluoromethoxy)phenylhydrazone
FCS: Fetal Calf Serum
HBSS: Hanks' Balanced Salt Solution
HC: Healthy Control
HE: Hepatic Encephalopathy
HUVEC: Human Umbilical Vein Endothelial Cell
IQR: Interquartile Range
ITS: Insulin Transferrin Selenium
LDA: Linear Discriminant Analysis
LPS: Lipopolysaccharide
LUMC: Leiden University Medical Centre
LUVDS: Leiden University Medical Centre Volunteer Donor Service
MELD: Model for End‐Stage Liver Disease
OCR: Oxygen Consumption Rate
PAMPs: Pathogen‐Associated Molecular Patterns
PBS: Phosphate‐Buffered Saline
PCA: Principal Component Analysis
PDP: Patient‐Derived Plasma
ROS: Reactive Oxygen Species
Rot/AA: Rotenone/Antimycin A
SBP: Spontaneous Bacterial Peritonitis
SPSS: Statistical Package for the Social Sciences
TNFα: Tumour Necrosis Factor Alpha
vWF: von Willebrand Factor

**Lay Summary**

This study demonstrates that plasma from patients with decompensated cirrhosis induces significant endothelial cell activation and mitochondrial alterations, which are partially restored by albumin supplementation. These findings are important to understand the cellular mechanisms driving complications in advanced cirrhosis and identify potential therapeutic targets beyond simply correcting hypoalbuminemia. Our ex vivo model may help identify patients who are most likely to benefit from albumin therapy and guide personalised treatment strategies. Future research is needed to identify the specific circulating factors driving mitochondrial damage and validate these findings in clinical studies.

## 1 Introduction

Cirrhosis is a chronic and progressive liver disease which arises from various etiological factors and is associated with a range of systemic manifestations [1]. Cirrhosis causes increased intrahepatic vascular resistance and, ultimately, portal hypertension. Decompensated cirrhosis is characterised by systemic inflammation and circulatory dysfunction, which affect multiple organs and systems and lead to complications including ascites, bacterial infections, gastrointestinal haemorrhages and hepatic encephalopathy (HE) [2].

As a result of impaired hepatocellular function, albumin synthesis by the liver is reduced in cirrhosis [3]. In advanced stages, hypoalbuminemia is further aggravated by ascites and renal dysfunction. Ascites develops in response to portal hypertension‐induced splanchnic vasodilation and subsequent sodium and water retention, contributing to dilutional hypoalbuminemia through plasma volume expansion and fluid shifts [4, 5]. Concurrently, renal dysfunction, often secondary to circulatory disturbances in advanced cirrhosis, can lead to increased urinary albumin losses and impaired tubular reabsorption, further lowering serum albumin levels [6].

Beyond its role in maintaining colloid osmotic pressure, albumin exerts a wide range of non‐oncotic functions that are essential to physiological homeostasis. These include antioxidant activity, scavenging of reactive species, immune modulation, binding and transport of endogenous and exogenous substances, and preservation of endothelial integrity [5, 6, 7, 8]. Previous studies have shown that serum albumin levels greater than 30 g/L are associated with a reduced inflammatory response in acute decompensation (AD) and acute‐on‐chronic liver failure (ACLF) [7].

Endothelial cell (EC) dysfunction is a critical driver of disease progression in patients with liver cirrhosis, contributing to acute decompensation (AD) and acute‐on‐chronic liver failure (ACLF). Endothelial cells (EC) line the lumen of the blood‐ and lymphatic vessels and act as a barrier that separates the blood from the interstitium, regulate vessel permeability, vascular tone, and the diapedesis of immune cells during inflammation [9, 10, 11]. In response to inflammatory and hemodynamic stressors, ECs can become activated, resulting in the loss of the EC barrier integrity and EC glycocalyx, coupled with upregulated expression of adhesion molecules, cytokines, and procoagulant factors [11, 12]. In advanced stages of cirrhosis, systemic EC activation is driven by pro‐inflammatory circulating factors such as pathogen‐associated molecular patterns (PAMPs) from bacterial translocation from the intestines, damage‐associated molecular patterns (DAMPs) from damaged, apoptotic, necroptotic or necrotic hepatocytes, and reactive oxygen species (ROS) and other toxins [12, 13, 14, 15]. This activated endothelial phenotype has been identified as a significant contributing factor to circulatory alterations in cirrhosis, resulting in the progression of portal hypertension and increased intrahepatic resistance [9, 16]. Furthermore, the severe EC activation driven by the systemic inflammation in decompensated cirrhosis and ACLF is thought to be a key driver for the progression into organ failure [17, 18].

Albumin deficiency further aggravates systemic EC activation driven by inflammatory mediators in advanced stages of cirrhosis. Experimental studies in cirrhotic rat models have shown that hypoalbuminemia has detrimental effects on EC function [19]. Conversely, human albumin showed to have protective effects on cultured human ECs upon lipopolysaccharide (LPS) activation by albumin endocytosis, thereby potentially acting as an intracellular ROS scavenger [19].

Recently, we developed a high‐throughput, image‐based assay to assess morphological responses of ECs to patient‐derived plasma (PDP) exposure [20]. Our previous findings indicated that peripheral plasma from cirrhotic patients triggers EC activation, and that the degree of EC activation and mitochondrial stress are closely correlated with the severity of the disease. In the current study, we aim to use this methodology to study the effects of albumin supplementation on EC morphological response to patient‐derived plasma exposure. We hypothesise that EC activation and mitochondrial stress will be decreased by the addition of albumin to reach physiological levels.

## 2 Methods

### 2.1 Patient Cohort

Patients were enrolled at the Leiden University Medical Centre (LUMC), a tertiary referral centre with liver transplantation facilities. Patients were eligible for inclusion if they had a confirmed diagnosis of cirrhosis based on histology, imaging, and/or laboratory results. Twenty consecutive patients with compensated cirrhosis and serum albumin levels > 30 g/L, and twenty consecutive patients with decompensated cirrhosis and hypoalbuminemia (serum albumin levels < 30 g/L) and without prior albumin suppletion in the 7 days prior to sampling were included. Decompensation was defined as having one or more of the following symptoms: Overt HE, gastrointestinalhaemorrhage, clinically manifest ascites grade 2 or 3 (according to the International Ascites Club) [21] or spontaneous bacterial peritonitis (SBP). Patients were excluded if they had received albumin within the 7 days prior to sampling.

EDTA plasma samples were collected from all included patients, and plasma samples from twenty healthy controls (HCs) were obtained by the Leiden University Medical Centre Volunteer Donor Service biobank (LUVDS). All patients and donors provided written informed consent. The study protocol conformed to the ethical guidelines of the 1975 Declaration of Helsinki and was approved by the local ethics committee (registration number RP24.019).

Clinical data were extracted from electronic patient medical records. Baseline characteristics regarding the aetiology of cirrhosis, comorbidities and use of medication were collected. Disease severity data were collected on the presence of infection, laboratory findings, Model for End‐Stage Liver Disease (MELD) score, Child Pugh class, Chronic Liver Failure Consortium Acute Decompensation (CLIF‐AD) score, and decompensating events as defined above, and laboratory values were collected. ACLF was defined according to EF‐CLIF criteria [22]. Infection was defined by one or more of the following criteria: A polymorphonuclear cell count in ascitic fluid of ≥ 250/mm^3^, a positive ascitic fluid culture, a positive blood culture, a positive urine culture or lesions on chest radiography suggestive of pneumonia.

### 2.2 Cell Culture

Human Umbilical Vein Endothelial Cells (HUVECs) were acquired from Lonza (C2517A). HUVECs were thawed and cultured in Endothelial cell growth medium 2 (EGM‐2) (PromoCell C‐22111) supplemented with 1% Penicillin/Streptomycin (Gibco 15 070–063) in a gelatin (1% in PBS, Merck; 104 078) coated T75 flask 3 days prior to seeding in a gelatin‐coated 96‐wells plate (PerkinElmer PhenoPlate, 6 055 300). Twenty thousand cells were seeded in each well, and cells were maintained in EGM‐2 for 5 days until plasma/cytokine exposure to allow a cobblestone‐like monolayer to form.

### 2.3 Plasma Exposure

Low passage (*p* = 2), single‐donor, confluent HUVEC monolayers were exposed to 25% recalcified K_2_EDTA plasma derived from either patients or HCs for 18 h. K_2_EDTA plasma was stabilised and recalcified by adding 0.5 μM recombinant Hirudin (ABCAM ab201396), 25 μg/mL Corn trypsin inhibitor (HTI CTI‐01), and 1.85 mM calcium‐dichloride (CaCl2) (Merck 1.02381) to a K_2_EDTA plasma volume equaling 25% of the total volume [20]. Endothelial cell basal medium (EBM) (PromoCell C‐22211) containing 1:100 insulin transferrin selenium supplement (ITS) (Gibco 41 400) was added to obtain the 90% of the volume. 37.5 g/L of a human albumin solution (200 g/L in 0.6% NaCl, Baxalta) or an equal volume 0.6% NaCl(Sigma Aldrich S9888) in water was added to the basal medium, and added in a 1:10 ratio to the plasma mix. Salt controls were added to all samples. Albumin solution was added to decompensated patient plasma to reach physiological albumin concentrations and to healthy volunteer plasma samples to reach supraphysiological levels. Three replicate wells were exposed for each plasma sample.

### 2.4 LPS, Bilirubin, and TNFα Exposure

Low passage (*p* = 2), single‐donor, confluent HUVEC monolayers were exposed to a titration series of LPS from *Escherichia coli* O111:B4 (Sigma Aldrich L4391‐1MG) (2 000 ng/mL, 200 ng/mL, 20 ng/mL, 2 ng/mL), TNFα (Sigma Aldrich H8916‐10UG) (1 ng/mL, 0.1 ng/mL, 0.01 ng/mL), or bilirubin (Sigma Aldrich 14 370‐250MG) (0.13 mg/mL, 0.013 mg/mL, 0.0013 mg/mL) in EBM + 1% Pen/Strep +5% FCS + 1:100 ITS supplement. Albumin in different concentrations: 20 g/L, 4 g/L, 0.8 g/L, and 0 g/L was added to the 200 ng/mL LPS condition, the 0.1 ng/mL TNFα condition, and the 0.013 mg/mL bilirubin condition. These conditions were chosen as previous experiments showed these concentrations were around the halfway point on the morphological dose response curves. The other conditions were not studied with exogenous albumin exposure.

### 2.5 Immunofluorescent Staining and Imaging

Cells were stained according to the previously published protocol, modified to include a staining for von Willebrand factor [20]. Cells were incubated with MitoTracker Deep Red FM (InVitrogen M22426) 1:2000 for 30 min, followed by incubation with 4% paraformaldehyde (Alfa Aesar J61899) for 10 min, and 30 min incubation with blocking buffer (2% (w/v) BSA + 0.5% Glycine (Merck 1.04201) + 1% (v/v) Triton X‐100 in PBS). Afterwards, wells were incubated with primary antibodies against VE‐Cadherin (BD 555661, 2 μg/mL) and vWF (DAKO a0082, 1:1000) in PBS with 2% BSA for one hour, followed by incubation with 488 Alexa‐fluorophore labelled Donkey anti‐mouse antibody (Invitrogen A‐11001, 2 μg/mL), Qdot 625 labelled Goat anti‐rabbit antibody (Invitrogen A‐10194, 1:500), 1:500 Rhodamine Phalloidin (Invitrogen R415), and 1:1000 HOECHST 33 258 (Molecular Probes) in 50 mM Borate Buffer, pH = 8.3 with 2% BSA + 0.05% Glycine for one hour. Wells were washed with borate buffer and stored under borate buffer for imaging. Max‐projections of 9 z‐steps with 0.7 μm step size were acquired using a high content confocal microscope (Molecular Devices, ImageXpress Micro Confocal) at 20× magnification (Nikon Plan Apo Lambda; NA = 0.75), using a 60 μm pinhole. Six sites without overlap were imaged per well.

### 2.6 Image Analysis

Single‐cell morphological profiles were extracted from the images using R (version 4.1.1) [23], the R‐package EBImage (version 4.29.2) [24], and in‐house scripts. In short, individual nuclei were identified from DAPI‐channel images and used as seeds to identify the cell borders using the VE‐Cadherin signal. To filter out debris, upper and lower thresholds were applied for the nuclei size. R‐package EBImage “compute” functions were used to compute morphological features. Functions to extract distribution and colocalisation were based on the procedure by Verbeek et al. [25].

### 2.7 Mitochondrial Mass, Membrane Potential, Superoxide Production, and Respiration

Mitochondrial mass, membrane potential, and superoxide production were determined with the fluorescent dyes MitoTracker Green FM, MitoTracker Red FM, and MitoSOX red, respectively. HUVECs were cultured, seeded into a 96‐well plate, and exposed to 25% pooled plasma from either healthy controls or patients with decompensated cirrhosis as described above. Following overnight incubation, wells were washed with warm HBSS (Gibco 14 025–050) and incubated for 45 min with HBSS containing 1:20000 MitoTracker Deep Red FM (InVitrogen M22426), 1:2000 MitoTracker Green (InVitrogen M7514), and 2.5 μM MitoSOX Red (InVitrogen M36008). After incubation, wells were washed 3 times with warm EBM + 2% FCS and incubated with EBM + 2% FCS for 45 min. Mitochondria were imaged using max‐projections of 5 z‐steps with 0.7 μm step size on a high content microscope (Molecular Devices, ImageXpress Micro Confocal) at 40× magnification (NA = 0.95), using a 60 μm pinhole and a heated, CO2‐ enriched, and humidified sample stage. The MitoSOX signal was normalised to the Mitochondrial potential as measured by the MitoTracker Red FM signal. Four sites without overlap were imaged per condition.

Mitochondrial respiration was measured using the Agilent Seahorse XF Cell Mito Stress assay. Six thousand HUVECs were seeded per well in a gelatin‐coated 96‐well Seahorse XF Cell Culture Microplate and cultured for 2 days in a humidified incubator at 37°C. Cells were then exposed to patient‐derived plasma from either healthy controls or patients with decompensated cirrhosis for 18 h as described above. Afterwards, the Seahorse XF Cell Culture Microplate was placed in the Agilent SeahorseAnalyser. Cells were exposed to 6 mM Glucose, 1.5 μM Oligomycin, 1 μM FCCP, and 0.5 μM Rot/AA. Oxygen consumption rate (OCR) and extracellular acidification rate (ECAR) were measured during the course of the experiment. Results were normalised to the number of cells present in the wells.

### 2.8 Data Analysis

Data analysis of the morphological data was performed in Python version 3.7.6, using functions from the Scikit‐learn package [26]. The approach was as follows: Z‐score normalisation was applied to the single‐cell data of each plate dataset separately [27]. Afterwards, datasets were combined. Single‐cell profiles were reduced in dimension by factor analysis [28], capturing about 80% of total variance, resulting in 11 factors. Mean profiles were created by averaging the cells per well for each patient/time point in the reduced subset. Resulting profiles were scaled to 0‐mean and unit variance.

Resulting profiles were further reduced in dimension by linear discriminant analysis by computing a model on the non‐albumin supplemented conditions, followed by scoring of the entire dataset. The conditions were compared by an independent *t*‐test. Conditions were compared for each Factor using the Wilcoxon signed‐rank test with FDR correction.

Statistical analyses of the clinical data were performed with IBM SPSS Statistics (Statistical Package for the Social Sciences, version 29.0). Baseline characteristics were presented as median and interquartile range (IQR) for continuous variables, and as frequencies and percentages for categorical variables. Differences between groups for continuous variables were assessed using the Mann–Whitney U test due to the non‐normal distribution of the data. Categorical variables were analysed using the Chi‐square test or Fisher's exact test when expected cell counts were less than five. A *p*‐value < 0.05 was considered statistically significant.

## 3 Results

### 3.1 Patient Characteristics

Patient characteristics are summarised in Table 1. The majority of patients in both groups were female 55% in the compensated cirrhosis (CC) group and 60% in the decompensated cirrhosis (DC) group. The median age was 57 years in the CC group and 62 years in the DC group. There were no significant differences in cirrhosis aetiology between the two groups (*p* = 0.483). Patients with DC had significantly higher MELD and AD scores compared to those with CC (MELD 19 [13, 14, 15, 16, 17, 18, 19, 20, 21, 22, 23, 24] vs. 7 [7, 8, 9]; AD‐score: 52 [47–61] vs. 40 [37–44]; *p* < 0.001). In the DC group, ascites was the most common decompensating event (75%), followed by HE (50%). ACLF was reported in 20% of the decompensated patients. Serum albumin levels were significantly lower in the DC cohort, with a median concentration of 25 g/L [23, 24, 25, 26, 27, 28], compared to 42 g/L [39–44] in the CC group (*p* < 0.001).

**TABLE 1:** Patient characteristics.

Baseline characteristics	Compensated (*n* = 20) *n* (%)	Decompensated (*n* = 20) *N* (%)	*p*
Age (median · IQR)	57 [50–64]	62 [56–67]	0.527
Gender			1.000
Male	9 (45)	8 (40)	
Female	11 (55)	12 (60)	
Aetiology cirrhosis			0.483
Alcoholic related	2 (10)	6 (30)	
MASLD	6 (30)	6 (30)	
Viral hepatitis	1 (5)	2 (10)	
A1ATdeficiency	1 (5)	1 (5)	
AIH/PSC/PBC	9 (45)	5 (25)	
Cryptogenic/other	1 (5)	0 (0)	
Previous AD	0 (0)	14 (70)	**< 0.001**
Child Pugh Class			**< 0.001**
A	20 (100)	0 (0)	
B	0 (0)	7 (35)	
C	0 (0)	13 (65)	
MELD score (median · IQR)	7 [7–9]	19 [13–24]	**< 0.001**
Medical history			
HCC	4 (20)	1 (5)	0.235
Diabetes Mellitus	8 (40)	7 (35)	1.000
Cardiovascular disease	8 (40)	10 (50)	**0.041**
Chronic Kidney disease	1 (5)	0 (0)	1.000
IBD	2 (10)	0 (0)	0.487
Medication			
Diuretics	0 (0)	15 (75)	**< 0.001**
OAC	2 (10)	11 (55)	**0.006**
Betablockers	10 (50)	6 (30)	0.333
Statins	6 (30)	6 (30)	1.000
PPI	12 (60)	15 (75)	0.501
Corticosteroids	6 (30)	3 (15)	0.451
Immunosuppressants	6 (30)	1 (5)	0.091
Antibiotics	0 (0)	8 (40)	**0.003**
Albumin	0 (0)	0 (0)	1.000
Decompensating events			
Ascites	0 (0)	15 (75)	**< 0.001**
SBP/bacterial infection	0 (0)	7 (35)	**0.008**
Overt Hepatic Encephalopathy	0 (0)	10 (50)	**0.003**
GI‐bleed	0 (0)	2 (10)	0.487
CLIF‐C AD score (median · IQR)		52.4 [46.5–60.7]	
ACLF	0 (0)	3 (20)	0.106
Laboratory values (median · IQR)			
INR	1.1 [1.0‐1.1]	1.6 [1.4–1.8]	**< 0.001**
Creatinine (mmol/L)	62 [54–71]	69 [49–94]	0.752
Albumin (g/L)	42 [39‐44]	25 [23–28]	**< 0.001**
Bilirubin (mmol/L)	13 [11‐26]	100 [30‐214]	**< 0.001**
WBC (10^*9^/L)	5.4 [4.3‐7.3]	6.3 [4.7‐7.4]	0.343

*Note:* Statistically significant values are shown in bold.

*Abbreviations:* A1ATD, A1‐antitrypsin deficiency; ACLF, acute‐on‐chronic liver failure; AD, acute decompensation; AIH, autoimmune hepatitis; HCC, hepatocellular carcinoma; IBD, inflammatory bowel disease; INR, international normalised ratio; IQR, interquartile range; MASLD, metabolic dysfunction‐associated steatotic liver disease; MELD, Model For End‐Stage Liver Disease; OAC, oral anticoagulants; PBC, primary biliary cholangitis; PPI, proton pump inhibitors; PSC, primary sclerosing cholangitis; SBP, spontaneous bacterial peritonitis; WBC, white blood count.

### 3.2 Modulatory Effect of Albumin on EC Response

**FIGURE 1:**
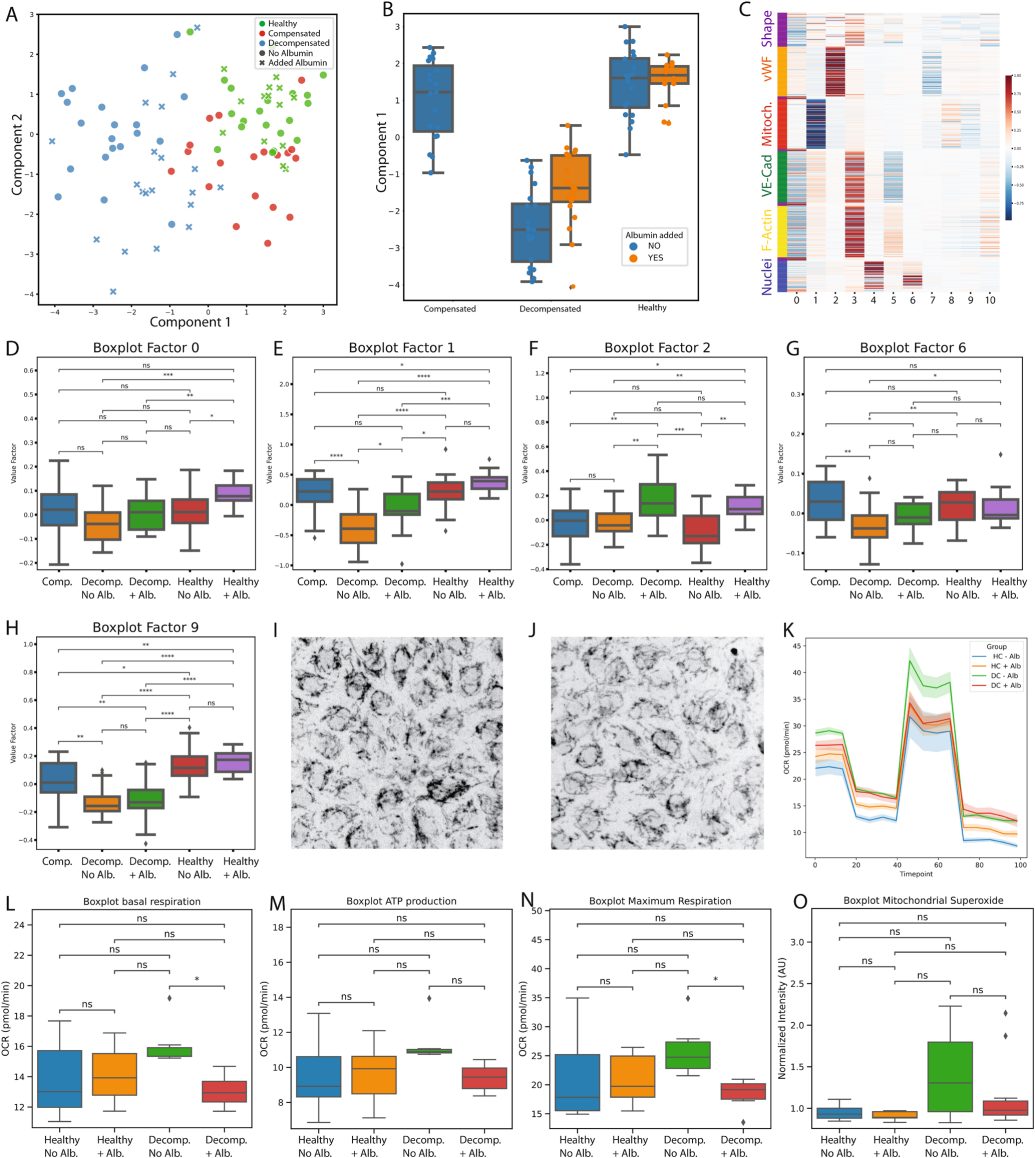
Supervised multivariate analysis of patient morphological profiles before and after albumin supplementation. (A) 2D LDA model score plot, fitted using the datapoints without albumin supplementation (round dots), and then used to score datapoints with added albumin (crosses). (B) Boxplot of the first component of the LDA model presented in A. (C) Regression coefficients for each factor are visualised to determine which cellular component was important in the construction of that Factor. More intense colours mean more weight in the construction of that Factor. (D–H) Factor values are plotted as boxplots for Factors that showed a significant difference between the groups. (I) Representative image of mitochondrial morphology of ECs exposed to plasma from decompensated patients without albumin supplementation. (J) Representative image of mitochondrial morphology of ECs exposed to plasma from the same patient as presented in E, with albumin supplementation. (K) Mitochondrial respiration changes measured by the Seahorse XF Cell Mito Stress assay. (L) Basal respiration rates of ECs exposed to plasma from the four groups. (M) ATP‐linked oxygen consumption rate (OCR). (N) Maximal respiration rates of ECs exposed to plasma from the four groups. (O) Superoxide production in the mitochondria of ECs after exposure to pooled patient plasma. Alb, albumin; comp, compensated cirrhosis; decomp, decompensated cirrhosis; LDA, linear discriminant analysis; VE‐Cad, VE‐cadherin; Mitoch, mitochondria; vWF, von Willebrand factor; F‐Actin, filamentous actin; ATP, adenosine triphosphate; OCR, oxygen consumption rate; ns, not significant.

ECs were exposed to plasma derived from patients with decompensated cirrhosis, compensated cirrhosis, and healthy controls. Multivariate analysis by the LDA model of the resulting morphological profiles showed that plasmas from different clinical groups elicit distinct morphological responses in the ECs (Figure 1A). To observe the effects of albumin in a diseased and non‐diseased state, albumin was added to the plasma from patients with DC and to the plasma from HC, reaching supraphysiological levels. 1D and 2D LDA models computed only on the EC responses to plasmas without added albumin were used to score the EC responses to plasmas with added albumin (Figure 1A,B). A large shift in EC morphology towards the healthy phenotype was observed for the patients with DC with added albumin, but no shift was observed for the HC upon the addition of albumin.

To link cellular components responsible for the observed shift in ECs exposed to plasma from patients with DC towards the healthier phenotype upon albumin supplementation, all Factors were investigated (Figure 1C–H). Information represented by the Factors can be traced back to the original cellular component, thereby aiding in biological interpretation (Figure 1C). Factor 1 was highly significant for the shift of patients with DC towards a healthy phenotype upon albumin supplementation (Figure 1E). This Factor contained morphological information about the mitochondria, as the regression coefficients were almost exclusively highest for mitochondrial features. Factor 2, representing information about vWF and Weibel‐Palade bodies, was found to be significant for effects by the albumin treatment itself (Figure 1F). Visual inspection of mitochondrial morphology further showed that ECs exposed to plasma from patients with DC suggested more fragmented mitochondria with increased signs of fission and a more pronounced perinuclearlocalisation, which appeared to be partially restored upon albumin supplementation (Figure 1I,J). OCR was increased for ECs exposed to plasma from patients with DC compared to HC (Figure 1K). Maximum respiration and basal respiration significantly decreased upon addition of albumin (Figure 1L,N). However, ATP production did not significantly decrease upon addition of albumin to plasma from patients with DC (Figure 1M). Most importantly, the addition of albumin to plasma from patients with DC lowered OCR to rates comparable with HC. Notably, the increase in mitochondrial respiration did not lead to a statistically significant increase in ROS production (Figure 1O).

### 3.3 Albumin Has No Modulatory Effect On EC Responses To TNFα or Bilirubin

**FIGURE 2:**
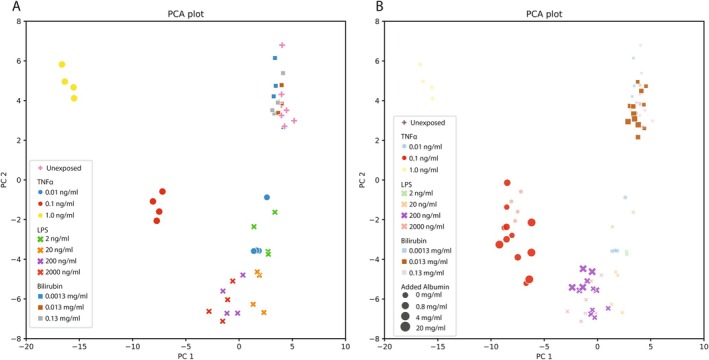
TNF‐α, LPS and bilirubin stimulation. (A) PCA score plot of the morphological profiles of ECs exposed to different concentrations of TNFα, LPS, and Bilirubin. (B) The model in A is used for scoring the effect of adding different concentrations of albumin to the LPS 200 ng/mL, TNFα 0.1 ng/mL, and the bilirubin 0.013 mg/mL conditions. All conditions with albumin supplementation show no shift in position in the PCA score plot compared to the baseline position (without added albumin). TNFα, tumour necrosis factor alpha; LPS, lipopolysaccharide; PCA, principal component analysis.

The modulatory effects of albumin on the EC responses were tested for well‐described circulating factors present in the plasma from patients with DC: TNFα, Bilirubin, and LPS. A titration series of these compounds was performed, with increasing albumin concentrations for a set concentration of TNFα, Bilirubin, or LPS. A PCA model was computed for the conditions without added albumin (Figure 2A). Clear dose‐dependent effects could be observed for TNFα and LPS; however, stimulation by bilirubin did not result in a different EC phenotype compared to unstimulated controls, as a clear overlap in the score plot is observed between this condition and the unstimulated. Scoring of the conditions with added albumin using this PCA model, Figure 2B, showed that no significant effect was observed for TNFα, bilirubin, or LPS supplemented with albumin.

## 4 Discussion

Our study demonstrates that plasma from patients with DC with hypoalbuminemia induces significant EC activation, characterised by alterations in cellular morphology, particularly mitochondrial morphology. Albumin supplementation mitigates these deleterious effects, restoring EC phenotype and mitochondrial morphology towards a healthier phenotype.

Mitochondrial morphology was significantly altered in response to stimulation by plasma from patients with DC compared to both CC and HC. This observation aligns with our previous work, showing mitochondrial fragmentation as a key morphological feature distinguishing advanced cirrhosis (CP‐C) from earlier cirrhosis stages (CP‐A and CP‐B) and healthy controls [20]. Mitochondrial dysfunction has emerged as a central component of systemic inflammation and disease progression in decompensated cirrhosis, contributing to impaired energy metabolism, oxidative stress, and organ failure [17]. However, its role in ECs has not been wellcharacterised. In the present study, we demonstrate that ECs exposed to plasma from patients with DC exhibit both morphological alterations and increased mitochondrial respiration, without a concomitant increase in ROS production. These findings suggest a state of increased mitochondrial activity rather than overt dysfunction, which may reflect an adaptive mitochondrial response to stressors present in PDP, such as pro‐inflammatory cytokines, metabolites and other factors. It would of interest to investigate whether prolonged or repeated exposure results in mitochondrial exhaustion and impaired function, although this was not assessed in the current experiment.

Multivariate analysis revealed that EC morphology is shifted towards a healthier phenotype upon albumin treatment for the decompensated group, and that this shift was strongly associated with the restoration of mitochondrial morphology. Importantly, cytoskeleton reorganisation and EC border remodelling were not restored by the albumin treatment, indicating that mitochondria are the key cellular component restored by albumin supplementation, and not the reduction of EC activation. These findings further support the hypothesis that mitochondrial dysfunction is not only a marker of disease severity but also a potential therapeutic target [29]. Mitochondrial fission phenotypes, commonly reported in systemic diseases such as cardiovascular conditions and diabetes mellitus, are associated with increased inflammatory responses, ROS production, and sustained NF‐κB activation [30, 31]. In the context of cirrhosis, this maladaptive response may exacerbate endothelial activation and contribute to disease progression [32].

Mitochondrial restoration by albumin treatment is likely due to its antioxidant properties, including its ability to act as an intracellular ROS scavenger and its capacity to modulate mitochondrial function [19, 33]. In our model, these effects occurred without a significant change in ROS production, suggesting that albumin may exert direct effects on mitochondrial function independent of ROS modulation. García‐Martínez et al. demonstrated that albumin exerts direct protective effects on ECs by reducing ROS production and reducing LPS‐induced activation [19]. They identified both extracellular mechanisms—such as binding LPS by albumin—and intracellular mechanisms via albumin endocytosis and antioxidant activity. Notably, albumin's beneficial effects were independent of oncotic pressure, pointing towards its non‐oncotic, pleiotropic functions in maintaining endothelial integrity. Considering the multiple biological actions of albumin, disentangling the mechanisms underlying the observed beneficial morphological and functional effects on endothelial cells is an important direction for future research, including studies comparing non‐oxidised and oxidised albumin to better define the contribution of its antioxidant properties.

Interestingly, while albumin supplementation showed a significant restorative effect on EC morphology in our patient plasma experiments, it had no significant effects against single inflammatory mediators such as LPS, TNFα, or bilirubin. These findings suggest that albumin's restoring effects are likely not by binding LPS or TNFα in plasma and preventing EC activation in this manner, but rather by restoring mitochondria from unknown toxic circulating factors. Despite observing low infection rates in the decompensated cirrhosis cohort, albumin supplementation improved endothelial and mitochondrial function, suggesting that albumin's mechanisms of action extend beyond infection control and likely involve direct modulation of cellular processes.

Previous clinical trials have investigated long‐term albumin administration in patients with decompensated cirrhosis, aiming to prevent complications and improve survival [7, 34, 35]. However, differences in study design and conflicting results suggest that not all patients are likely to benefit from albumin therapy. Our findings support this hypothesis, highlighting the need for a personalised approach based on biomarker‐driven patient stratification to identify those most likely to respond to treatment. In this context, the use of ex vivo EC‐based assays to stratify patients may have important implications for personalised medicine. Albumin supplementation could be a valuable therapeutic strategy to improve endothelial function and reduce the risk of complications in selected patients with decompensated cirrhosis.

### 4.1 Limitations and Future Perspectives

This study has some limitations that can be addressed in future research. First, the sample size was relatively small, which may limit the generalisability of the findings. The predictive accuracy of the assays can be refined in larger cohorts. Second, the utility of these assays for monitoring disease progression and response to therapy should be explored in future longitudinal studies. Third, although alterations in mitochondrial morphology and function were assessed, the underlying mechanisms and circulating factors driving mitochondrial dysfunction in decompensated liver cirrhosis remain to be elucidated and are areas for future research.

In conclusion, our study demonstrates that plasma from patients with decompensated cirrhosis induces significant EC activation, characterised by alterations in cellular morphology, particularly mitochondrial morphology and function. Albumin supplementation mitigates these deleterious effects, restoring EC phenotype towards a healthier state. However, further clinical trials are needed to definitively establish the efficacy and safety of albumin therapy in this patient population. Additionally, our in vitro model contributes to the identification of therapeutic targets and holds potential for predicting treatment responses in the context of endothelial activation in cirrhosis.

## Author Contributions

S.E.F., R.J.P., A.J.v.Z., and M.J.C. conceptualised and designed the study. R.J.P. conceptualised and performed the experimental methodology and data analysis. Patient selection, plasma collection, and clinical data analysis were performed by S.E.F. S.E.F. and R.J.P. contributed to the experiments, acquisition of data, interpretation of the data, and drafting the manuscript. A.J.C.K. and R.B. participated in the critical revision of the paper for important intellectual content. M.J.C. and A.J.v.Z. participated in the interpretation of the data, critical revision of the manuscript for important intellectual content, and study supervision.

## Funding

MC and SF received funding from the European Union's Horizon 2020 research and innovation program for MICROB‐PREDICT (project ID 825694). The funders had no role in study design, data collection and analysis, decision to publish, or preparation of the manuscript.

## Disclosure

The authors have nothing to report.

## Ethics Statement

All ethical standards were followed according to the revised Helsinki declaration. This study was ethically approved by the Medical Ethical Committee of the Leiden University Medical Centre (RP24.019). All patients and donors provided written informed consent.

## Conflicts of Interest

The authors declare no conflicts of interest.

## Data Availability

The data that support the findings of this study are available from the corresponding author upon reasonable request.
